# *CEACAM6* gene expression in intrahepatic cholangiocarcinoma

**DOI:** 10.1038/sj.bjc.6603276

**Published:** 2006-07-25

**Authors:** K Ieta, F Tanaka, T Utsunomiya, H Kuwano, M Mori

**Affiliations:** 1 Department of Surgery and Molecular Oncology, Medical Institute of Bioregulation, Kyushu University, 4546 Tsurumihara, Beppu, 874-0838, Japan; 2 Department of General Surgical Science, Graduate School of Medicine, Gunma University, 3-39-22 Showa-machi, Maebashi, Gunma, 371-8511, Japan

**Keywords:** carcinoembryonic antigen-related cell adhesion molecule 6 (*CEACAM6*), cholangiocarcinoma, gemcitabine

## Abstract

The purpose of this study was to investigate the clinicopathological and biological significance of human carcinoembryonic antigen-related cell adhesion molecule 6 (*CEACAM6*) gene expression in human intrahepatic cholangiocarcinoma. *CEACAM6* is reported to be involved in human malignancies. However, in cholangiocarcinoma expression of *CEACAM6* and its clinicopathological significance have not been investigated. *CEACAM6* expression status was determined and analysed with respect to various clinicopathological parameters in 23 intrahepatic cholangiocarcinomas. Additionally, we investigated effects of *CEACAM6* gene in the cholangiocarcinoma cell lines. *CEACAM6* gene expression in cancer tissues was higher than in noncancerous tissues in 16 of the 23 cases; however, it was not statistically significant. The tumours with elevated *CEACAM6* expression showed a tendency to be associated with lymphatic invasion and stage of the disease. Interestingly, patients with high *CEACAM6* expression showed a significantly poorer disease-free survival rate than those with low *CEACAM6* expression. We demonstrated that *CEACAM6*-transfected cells were more proliferative, more invasive and more chemoresistant to gemcitabine compared to mock-transfected cells. Furthermore, *CEACAM6* gene silencing by *CEACAM6*-specific siRNA resulted in higher chemosensitivity to gemcitabine. *CEACAM6* is a potential prognostic indicator and potential chemoresistant marker to gemcitabine for patients with intrahepatic cholangiocarcinoma.

Cholangiocarcinoma is a malignant neoplasm arising from the biliary epithelium, either within the liver (intrahepatic cholangiocarcinoma) or extrahepatic bile duct (extrahepatic cholangiocarcinoma). Although it is a relatively rare malignancy, the worldwide incidence of intrahepatic cholangiocarcinoma is steadily increasing (Olnes and Erlich, 2004; Khan *et al*, 2005; Lazaridis and Gores, 2005). The overall survival rate is poor; <5% of patients survive more than 5 years, and the rate has not changed significantly over the past 30 years (Shaib and El-Serag, 2004). The curative treatment of cholangiocarcinoma is surgical resection. More than two-thirds of patients with cholangiocarcinoma are not resectable at the time of initial diagnosis (Khan *et al*, 2002), because it is clinically silent until it has become an advanced disease with obstructive symptoms. Chemotherapy for cholangiocarcinoma is carried out for those patients who are inoperable or who have recurrent disease, but the results tend to be disappointing (Olnes and Erlich, 2004; Khan *et al*, 2005). Several different new anticancer drugs are under investigation for the treatment of advanced cholangiocarcinoma (Thongprasert, 2005). Among those, one agent – the nucleoside analog gemcitabine – was reported to show efficacy in treating advanced cholangiocarcinoma (Thongprasert, 2005).

Carcinoembryonic antigen-related cell adhesion molecule 6 (*CEACAM6*) is a glycosylphosphatidylinositol(GPI)-linked immunoglobulin super family member (Thompson *et al*, 1991) that is overexpressed in a variety of malignancies (Hasegawa *et al*, 1993; Kodera *et al*, 1993; Jantscheff *et al*, 2003; Duxbury *et al*, 2004a). Despite lacking an intracellular domain, *CEACAM6* is able to influence intracellular signaling events, and overexpression of this molecule appears to promote gastrointestinal cancer progression (Scholzel *et al*, 2000; Ilantzis *et al*, 2002). Silencing of the *CEACAM6* gene impairs metastasis and suppresses tumor growth (Duxbury *et al*, 2004a, 2004b). In cholangiocarcinoma, *CEACAM6* expression and its relation to clinicopathological factors have not been investigated.

In the present study, we investigated the expression of *CEACAM6* in patients with intrahepatic cholangiocarcinoma using real-time quantitative reverse transcription–polymerase chain reaction (RT-PCR) to analyse the association of clinicopathological factors and prognosis with *CEACAM6* expression levels. Furthermore, we established a *CEACAM6* stably transfected human cholangiocarcinoma cell line and examined the biological behaviour of *CEACAM6*-transfected cells, such as cell growth, invasiveness, resistance to anoikis and gemcitabine chemosensitivity. We also examined whether silencing the *CEACAM6* gene by siRNA enhanced gemcitabine chemosensitivity.

## MATERIALS AND METHODS

### Cell lines

The human cholangiocarcinoma cell lines, TFK-1, HuCC-T1 and MEC were provided by the Cell Resource Center of Biomedical Research, Institute of Development, Aging and Cancer (Tohoku University, Sendai, Japan) and maintained in RPMI 1640 medium containing 10% fetal bovine serum (FBS) and antibiotics at 37°C in a 5% humidified CO_2_ atmosphere.

### Clinical samples

Patients (23) with intrahepatic cholangiocarcinoma who underwent surgery at the Medical Institute of Bioregulation Hospital and the Department of Surgery and Science, Kyusyu University between 1993 and 2002 were enrolled in this study. All patients underwent a resection of the primary cancer. No patients received chemotherapy or radiotherapy prior to or after operation. The resected cancer (T) and paired noncancerous (N) tissue specimens were immediately frozen in liquid nitrogen and kept at −80°C until the extraction of RNA. Written informed consent was obtained from all patients. The follow-up ranged from 1 to 74 months with a median of 12 months.

### Oligonucleotide primers for *CEACAM6* gene amplification by RT–PCR

Total RNA was extracted from each sample and complementary DNA (cDNA) was synthesized from 8 *μ*g of total RNA using random hexamer primers and M-MLV reverse transcriptase (Invitrogen Crop., Carlsbad, CA, USA) as described previously (Mori *et al*, 2002).

The oligoribonucleotide primers for *CEACAM6* (226 bp) were sense primer: 5′-GAAATACAGAACCCAGCGAGTGC-3′; antisense primer: 5′-CAGTGATGTTGGGGATAAAGAGC-3′, glyceraldehyde 3-phosphate dehydrogenase (*GAPDH*) (270 bp) sense primer: 5′-TTGGTATCGTGGAAGGACTCA-3′; antisense primer: 5′-TGTCATCATATTTGGCAGGTT-3′. To avoid amplification of contaminating genomic DNA, these primers spanned more than two exons. The amplification was performed for 30 cycles of 1 min at 95°C, 1 min at 69°C and 1 min at 72°C. An 8 *μ*l aliquot of each PCR-amplified DNA was electrophoresed on a 2% agarose gel containing ethidium bromide. To ensure that the RNA was of sufficient purity for RT–PCR, a PCR assay with primers specific for the *GAPDH* gene was carried out in each case, except that only 22 cycles were performed under the following cycling conditions: 1 min at 95°C, 1 min at 56°C and 1 min at 72°C.

### Real-time quantitative RT–PCR

The PCR amplification for quantification of *CEACAM6* and *GAPDH* mRNA in clinical samples was performed in the LightCycler system (Roche Applied Science, IN, USA) using the LightCycler-FastStart DNA Master SYBR Green I kit (Roche Applied Science, IN, USA) as described previously (Ogawa *et al*, 2005). The amplification conditions of cycles consisted of initial denaturation at 95°C for 10 min, followed by 40 cycles of denaturation at 95°C for 10 s, annealing at 72°C (60°C for *GAPDH*) for 10 s, and elongation at 72°C for 10 s. For distinguishing specific from nonspecific products and primer dimers, melting curve analysis was carried out. To verify the melting curve results, each representative sample of the PCR products was electrophoresed on 2% agarose gels, and a single PCR product of the size predicted from the cDNA was confirmed. To quantitate the amount of specific mRNA in the samples, a standard curve was produced for each run measuring three points of the diluted TFK-1 cDNA. The concentrations of each sample were calculated by observing their crossing point to a standard curve. The relative expression levels of *CEACAM6* were obtained by normalizing the amount of *CEACAM6* mRNA divided by that of *GAPDH* mRNA as an endogenous control in each sample. Each assay was performed at least twice to verify the results, and the mean mRNA expression was used for analysis.

### Immunohistochemistry

Immunohistochemical studies of CEACAM6 were performed on surgical specimens from intrahepatic cholangiocarcinoma patients on formalin-fixed, paraffin-embedded tissues. After deparaffinization and blocking, the antigen–antibody reaction was incubated overnight at 4°C. ENVISION reagents (ENVISION+ Dual Link/HRP, Dako Cytomation, Denmark) were applied to detect the signal of the antigen-antibody reaction. All sections were counterstained with haematoxylin. The primary mouse monoclonal antibodies against CEACAM6 (GM7G5, Alexis Biochemicals, USA) were used at dilution of 1 : 100.

### Flow cytometry analysis

The cells were harvested and rinsed twice with PBS. Dissociated cells were stained with PE-conjugated anti-CEACAM6 antibody (KOR-SA3544 antigen-PE, Beckman Coulter, USA) and incubated for 30 min at room temperature. Cells (10 000) were collected for each sample using FACScan, and the data were analysed with CellQuest software (Becton Dikinson, San Jose, CA, USA).

### Cytotoxic assay

Cytotoxicity was determined by 3-(4, 5-dimethylthiazol-2-yl)-2, 5-diphenyl tetrazolium bromide (MTT) assay (Roche Diagnostics Corp., GmbH). Logarithmically growing cells were seeded at 3–5 × 10^3^ cells well^−1^ in microtitre plate wells (96 wells, flat bottom) in a final volume of 100 *μ*l culture medium per well, in a humidified atmosphere (37°C and 5% CO_2_). The cells were cultured overnight for adherence and gemcitabine was added at a concentration of 0–1 mg ml^−1^ into the plates. Gemcitabine-induced cytotoxicity was determined after 48–72 h of exposure. After incubation, 10 *μ*l of MTT labelling reagent (final concentration 0.5 mg ml^−1^) was added to each well. The microtitre plate was incubated for 4 h in a humidified atmosphere. Solubilisation solution (100 *μ*l) was added to each well. The plate was allowed to stand overnight in the incubator in a humidified atmosphere. After checking for complete solubilisation of the purple formazan crystals, the spectrophotometrical absorbance of the samples was measured using a model 550 microplate reader (Bio-Rad Laboratories, CA, USA), at a wavelength of 570 nm corrected to 655 nm. Each independent experiment was performed three times.

### Transfection assays and establishment of a stable *CEACAM6*-transfected cholangiocarcinoma cell line

Human *CEACAM6* cDNA was generated by RT–PCR and subcloned into pcDNA3.1/Hygro^©^ (+) expression vector (Invitrogen Corp., Carlsbad, CA, USA) according to the manufacturer’s protocol. To confirm accurate insertion into the frame of the expression vector, sequencing chemistry were performed. Transfection into the cholangiocarcinoma cell line (HuCC-T1) was performed by the Lipofectamine™2000 method (Invitrogen Crop., Carlsbad, CA, USA) as described previously (Shibata *et al*, 1999). Then, stable transfected clone expressing abundant *CEACAM6* protein were selected after hygromicine B (600 *μ*g ml^−1^) (Invitrogen Crop., Carlsbad, CA, USA) treatment and used for the subsequent experiments. A mock vector-transfected clone was used for control.

### *In vitro* proliferation assay

#### MTT assay

Logarithmically growing cells were seeded at 5 × 10^3^ cells well^−1^ in microtiter plate wells (96 wells, flat bottom) in a final volume of 100 *μ*l culture medium per well. After 0–96 h culture, spectrophotometrical absorbance of the samples was measured as described above. Each independent experiment was performed three times.

#### ELISA analysis

ELISA was performed using the BrdU ELISA kit (Roche Diagnostic Corp., Tokyo, Japan). Logarithmically growing cells were seeded at 1 × 10^4^ cells well^−1^ in microtitre plate wells (96 wells, flat bottom) in a final volume of 100 *μ*l culture medium per well. After overnight culture, BrdU was added to a concentration of 10 *μ*M into each well and incubated for 5 h. Cells were fixed by FixDenat and anti-BrdU-POD reaction liquid was added. The spectrophotometrical absorbance of the samples was measured using a model 550 microplate reader (Bio-Rad Laboratories, CA, USA), at a wavelength of 450 nm. Each independent experiment was performed three times.

### Cell cycle analysis

Cells (1.0 × 10^6^) were preincubated for 48 h or 72 h in serum-free medium at 37°C and then were kept in medium with serum (10% FBS) for 18 h at 37°C. The cells were harvested and fixed in 70% ethanol at −20°C. Then, the cells were washed and resuspended in propidium iodide (PI) staining buffer (5 *μ*g ml^−1^ PI and 0.25 mg ml^−1^ RNase) in PBS. DNA content was evaluated using an EPICS XL flow cytometer (Beckman Coulter Corp., Tokyo, Japan).

### *In vitro* invasion assay

*In vitro* invasion assays were performed using the BD BioCoat™ Tumor Invasion System (Becton Dickinson, San Jose, CA, USA) to evaluate invasive cells as described previously (Albini *et al*, 1987). Cells (5.0 × 10^4^ cells well^−1^) were placed in the upper chamber, and the lower chamber was filled with 750 *μ*l of RPMI1640 with 10% FBS as a chemoattractant. After 48 h and 72 h of incubation at 37°C, the membranes were labelled with Calcein-AM solutions. The invasive cells that had migrated through the membrane to the lower surface were read in a fluorescence plate reader at excitation/emission wavelengths of 485/530 nm using Multilabel Plate Counters VICTOR3 (PerkinElmer, Inc., USA).

### Anoikis assay

Anoikis was induced by poly (2-hydroxyethyl methacrylate) (poly-HEMA, Sigma) culture. A solution of 120 mg ml^−1^ poly-HEMA in 100% ethanol was made and diluted 1 : 10 in 95% ethanol. Of this solution 0.95 *μ*l was pipetted into 35 mm wells and left to dry for 48 h at room temperature. Prior to use, the wells were washed twice with PBS and once with RPMI-1640. Cells (1 × 10^6^), suspended in 2 ml of RPMI-1640 with 10% FBS, were incubated in the poly-HEMA-coated wells for 18 h in a humidified (37°C, 5% CO_2_) incubator. Following the induction of anoikis, the cells were washed and resuspended in 0.5 ml of binding buffer, and annexin V/fluorescein isothiocyanate/propidium iodide labelling was performed in accordance with the manufacturer's protocol (BD biosciences). Analysis was performed by FACScan. A total of 10 000 cells were collected for each sample, and the data were analysed with CellQuest software (Becton Dickinson, San Jose, CA, USA).

### *CEACAM6* RNA interference

*CEACAM6*-specific siRNA (Silencer™ Predesigned siRNA; sense: GGAGGUUCUUCUACUCGCCtt, antisense: GGCGAGUAGAAGAACCUCCtt) and negative control siRNA (Silencer™ Negative Control siRNA) were purchased from Ambion, USA. Logarithmically growing cells (TFK-1) were seeded at either 1.5 × 10^5^ or 3 × 10^3^ cells well^−1^ in a final volume of 2 ml or 100 *μ*l in six- or 96-well flat-bottom microtitre plates, respectively. The cells were cultured overnight for adherence. siRNA oligomer was diluted with Opti-MEN® I Reduced Sereum Medium (Invitrogen Corp., Carlsbad, CA, USA) and incubated for 5 min at room temperature. The diluted siRNA oligomer was mixed with the diluted Lipofectamine™2000 and incubated for 20 min at room temperature to allow siRNA-Lipofectamine™2000 complexes to form. The siRNA-Lipofectamine™2000 complexes were added to each well to a final concentration of 50 pmol ml^−1^. The cells were incubated in humidified atmosphere (37°C and 5% CO_2_) and the growth medium was replaced after 4 h. The assay was performed after 48 h incubation.

### Statistical analysis

For continuous variables, the data were expressed as the means±standard deviation (s.d.). The relationship between the *CEACAM6* expression and clinicopathological factors and *in vitro* assay data were analysed using the Student's *t* test, the Wilcoxon/Kruskal–Wallis test, *χ*^2^ test, and Repeated Measures ANOVA analysis. Overall survival curves and disease-free survival curves were plotted according to the Kaplan–Meier method measured from the day of surgery, and the generalised Wilcoxon test and the log-rank test were applied for comparison. All differences were deemed significant at the level of *P*<0.05. Statistical analysis was performed with the JMP software package (SAS Institute Inc., Cary, NC, USA).

## RESULTS

### Clinical study

#### Expression of *CEACAM6* mRNA in cell lines and clinical tissue specimens

**Figure 1:**
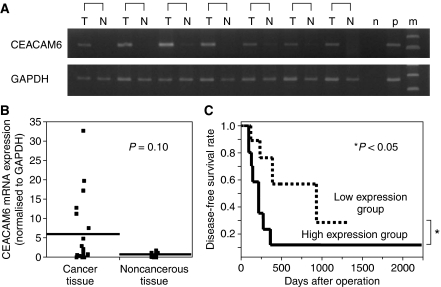
(**A**) Expression of *CEACAM6* by RT–PCR in representative intrahepatic cholangiocarcinoma patient tissues (T: cancer tissue, N: noncancerous tissue, n: negative control, p: positive control, m: indicates marker). (**B**) *CEACAM6* mRNA expression in cancer and noncancerous tissue with intrahepatic cholangiocarcinoma patients by real-time PCR (*n*=23). Horizontal lines indicate means. (**C**) Kaplan–Meier disease-free survival curves in patients with intrahepatic cholangiocarcinoma according to the level of *CEACAM6* mRNA expression. The recurrence rate for patients in the high expression group was significantly higher than that for patients in the low expression group (*P*<0.05). High expression group (*n*=10): *CEACAM6*/*GAPDH* ⩾2, Low expression group (*n*=13): *CEACAM6*/*GAPDH* <2.

Expression of *CEACAM6* mRNA in cell lines was shown by RT–PCR. TFK-1 cells showed a higher expression level of *CEACAM6* mRNA than that of HuCC-T1 and MEC cells. The expression of *CEACAM6* mRNA in cancer and noncancerous tissues of the patients with intrahepatic cholangiocarcinoma was examined by RT–PCR and real-time PCR. The expression levels of *CEACAM6* mRNA, which were corrected for those of *GAPDH* mRNA, in cancer tissues (5.90±8.74; mean±s.d.) were higher than in noncancerous tissues (0.70±0.48; mean±s.d.) in 16 of the 23 cases (69.6%). No differences in mRNA mean expression level was found between cancer and noncancerous tissues statistically (*P*=0.10: the Wilcoxon/Kruskal–Wallis test) (Figure 1A and B). The cases with values of *CEACAM6* under 2 in cancer tissues were considered to be a low expression group (*n*=13), whereas those with values 2 or over were considered to be a high expression group (*n*=10). The clinical implications of *CEACAM6* in patients with intrahepatic cholangiocarcinoma were evaluated by comparisons between these two groups.

### The clinicopathological significance of *CEACAM6* mRNA expression in intrahepatic cholangiocarcinoma

The clinicopathological features analysed in relation to the *CEACAM6* expression status are given in Table 1. The clinicopathological factors showed a tendency that the high expression group (seven of 10, 70.0% lymphatic invasion) showed more invasiveness of lymph nodes compared to the low expression group (four of 13, 30.8% lymphatic invasion) (*P*=0.06). The high expression group (six of 10, 60.0% stage 4) was also at a later progression stage of disease than the low expression group (three of 13, 23.0% stage 4) (*P*=0.09). On the other hand, no significant differences were observed regarding age, gender, tumour size, lymph node metastasis, vascular invasion, perineural invasion or histology. In the disease-free survival curve, patients in the high expression group had a significantly poorer prognosis than those in the low expression group, as illustrated in Figure 1C (*P*<0.05; Wilcoxon test, log-rank test). The overall survival rate between the two groups was not statistically different (*P*=0.84; log-rank test) (data not shown).

### Immunohistochemical staining

**Figure 2:**
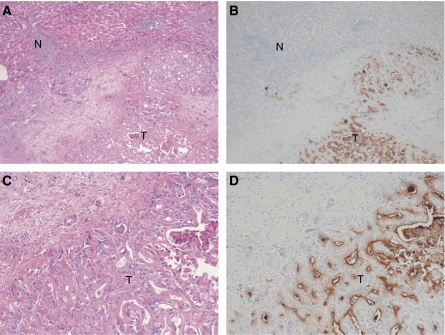
Immunohistochemistry with CEACAM6 antibody in intrahepatic cholangiocarcinoma. The majority of stain was observed in cancer cells. (**A**) original magnification × 40, haematoxylin and eosin stain, (**B**) original magnification × 40, CEACAM6 stain, (**C**) original magnification × 100, haematoxylin and eosin stain, (**D**) original magnification × 100, CEACAM6 stain, T: cancer tissue, N: noncancerous tissue.

Staining of CEACAM6 was markedly stronger in cancer tissue than corresponding noncancerous hepatic tissue. Expression of CEACAM6 was localized to the cell membrane (Figure 2). The immunohistochemical results closely corresponded with the RT–PCR.

### Experimental study

#### Chemosensitivity of gemcitabine in cholangiocarcinoma cell lines

**Figure 3:**
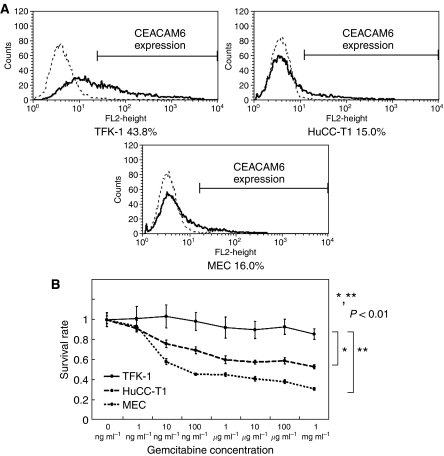
(**A**) Expression of CEACAM6 in three cholangiocarcinoma cell lines (TFK-1, HuCC-T1, MEC) by flow cytometry. TFK-1 cells showed higher expression of CEACAM6 than HuCC-T1 and MEC. (**B**) Gemcitabine chemosensitivity of cholangiocellular carcinoma cell lines. After 48 h gemcitabine exposure, survival rates of each cells were measured by MTT assay. TFK-1 cells were more chemoresistant than HuCC-T1 and MEC (*P*<0.01). The data represent the mean±s.d.

Flow cytometry analysis of expression levels of the *CEACAM6* protein in cholangiocarcinoma cell lines showed that TFK-1 cells had higher expression levels of the *CEACAM6* protein than the other two cell lines (HuCC-T1, MEC) (Figure 3A). The expression levels of *CEACAM6* protein was correlated with that of mRNA. We analysed the relation between *CEACAM6* expression and gemcitabine sensitivity in cholangiocarcinoma cell lines. MTT assays for sensitivity of gemcitabine showed TFK-1 cells were more resistant than HuCC-T1 and MEC cells (*P*<0.01: Repeated Measures ANOVA analysis) (Figure 3B). TFK-1 cells that highly expressed *CEACAM6* were more resistant to gemcitabine than other cell lines.

### The effect of *CEACAM6* gene transfected into the cholangiocarcinoma cell line

**Figure 4:**
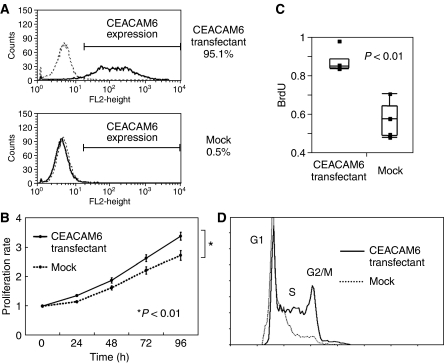
(**A**) CEACAM6 Expression of transfectants and mock cells (HuCC-T1). Expression levels of CEACAM6 protein were markedly increased in transfectants. (**B**) MTT assay. *CEACAM6*-transfectants were more proliferative than mock cells (*P*<0.01). The data represent the mean±s.d. (**C**) Comparison of BrdU uptake. The average BrdU uptake of transfectants was increased compared with mock cells. The data represent the mean±s.d. (**D**) Cell cycle analysis of transfectants and mock cells. After 48 h starvation and 18 h incubation with serum, transfectants (73.1%) were more in S phase than mock cells (63.4%).

HuCC-T1 cells showed a low expression level of *CEACAM6*. Stable *CEACAM6* overexpressing clones were established using the pcDNA3.1/hygro^©^ (+) expression vector. Overexpression of *CEACAM6* was confirmed with RT–PCR and flow cytometry (Figure 4A).

We analysed whether overexpression of *CEACAM6* would alter the growth rate of HuCC-T1 cholangiocarcinoma cells. As shown in Figure 4B and 4C, there was a significant difference in growth rate between the *CEACAM6* overexpressing cells and the mock-transfected cells (MTT assay: *P*<0.01; Repeated Measures ANOVA analysis, ELISA analysis: *P*<0.01; Student's *t* test). In cell cycle analysis, after 48 h serum starvation and 18 h incubation with serum, a greater percentage of *CEACAM6* overexpressing cells (73.1% S phase) were in S phase than mock-transfected cells (63.4% S phase) (Figure 4D). After 72 h serum starvation, the same pattern was identified.

**Figure 5:**
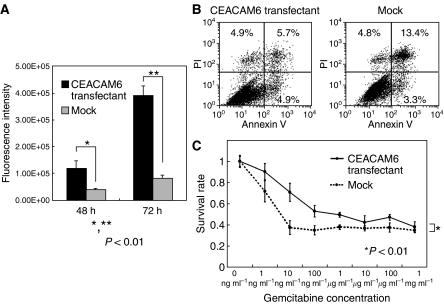
(**A**) Invasion assay. The invasiveness of transfectants was significantly stronger than that of mock cells (*P*<0.01). The data represent the mean±s.d. (**B**) Anoikis analysis. After anoikis induction for18 h, the apoptosis rates were measured by Annexin V and PI staining. Apoptosis cells were calculated as UR+LR (transfectant: UL: 4.9%, UR: 5.7%, LL: 84.5%, LR: 4.9%, mock: UL: 4.8%, UR: 13.4%, LL: 78.5%, LR: 3.3%). Proportion of apoptotic cells in transfectants (10.6%) was less than mock cells (16.7%). (UL: upper left, UR: upper right, LL: lower left, LR: lower right) (**C**) Gemcitabine chemosensitivity of transfectants and mock cells. After 72 h of gemcitabine exposure, survival rates were measured by MTT assay. Transfectants were more chemoresistant than mock cells (*P*<0.01). The data represent the mean±s.d.

In the clinicopathological studies, we found a tendency that the incidence of lymphatic invasion was higher in the *CEACAM6* high expression group than in the *CEACAM6* low expression group (Table 1). To verify these findings in an *in vitro* assay, we examined the invasive potential of the *CEACAM6* overexpressing cells using an *in vitro* matrigel invasion assay. Invasion assay showed that *CEACAM6* overexpressing cells were more significantly invasive than mock-transfected cells (*P*<0.01: the Wilcoxon/Kruskal–Wallis test) (Figure 5A). High expression of *CEACAM6* enhanced tumour invasiveness.

Anoikis is associated with cellular invasion and metastatic potential. After anoikis-induced culture, mock-transfected cells (16.7%) were more apoptotic than *CEACAM6* overexpressing cells (10.6%). *CEACAM6* overexpressing cells were more resistant to anoikis than mock-transfected cells (Figure 5B).

We examined whether *CEACAM6* expression in cholangiocarcinoma cell line (HuCC-T1) would alter sensitivity to gemcitabine. We compared *CEACAM6* overexpressing cells and mock-transfected cells. Overexpression of *CEACAM6* induced chemoresistance to gemcitabine which was significantly increased as observed by the MTT assay (*P*<0.01: Repeated Measures ANOVA analysis) (Figure 5C).

### The effect of *CEACAM6* gene silencing on gemcitabine chemosensitivity

**Figure 6:**
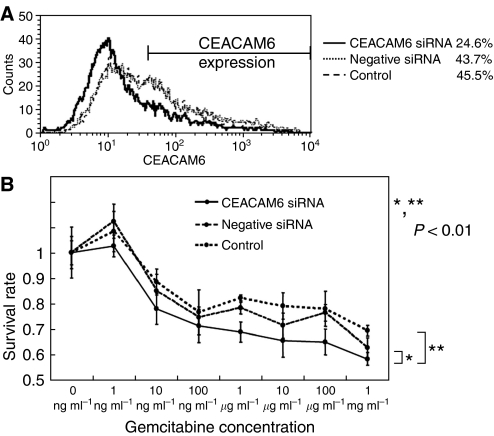
(**A**) CEACAM6 Expression of *CEACAM6* suppressed cells by *CEACAM6* specific-siRNA and control cells (TFK-1). 48 h after siRNA addition, expression of *CEACAM6* was measured by flow cytometry. (**B**) Gemcitabine chemosensitivity of *CEACAM6* suppressed cells and control cells. 72 h after gemcitabine exposure, survival rates were measured by MTT assay. *CEACAM6* suppressed cells were more chemosensitive to gemcitabine than mock cells (*P*<0.01). The data represent the mean±s.d.

TFK-1 cells showed a high expression level of *CEACAM6* and strong chemoresistance to gemcitabine. We examined whether suppression of *CEACAM6* expression would alter sensitivity to gemcitabine. The expression level of mRNA and protein was suppressed by *CEACAM6*-specific siRNA as confirmed by RT–PCR and flow cytometry analysis (Figure 6A). Suppression of *CEACAM6* expression reduced chemoresistance to gemcitabine (*P*<0.01: Repeated Measures ANOVA analysis) (Figure 6B).

## DISCUSSION

*CEACAM6* is highly expressed in various human cancer tissues and its clinical significance has been widely reported (Hasegawa *et al*, 1993; Kodera *et al*, 1993; Scholzel *et al*, 2000; Ilantzis *et al*, 2002; Jantscheff *et al*, 2003; Duxbury *et al*, 2004a). *CEACAM6* overexpression independently predicts poor overall and disease-free survival, and correlates inversely with cellular differentiation in colorectal cancer (Ilantzis *et al*, 2002; Jantscheff *et al*, 2003). Duxbury *et al* (2005) showed that *CEACAM6* expression was associated with adverse pathologic features and prognosis in pancreatic cancer. In gastric cancer, *CEACAM6* was overexpressed; however, there was no significant association between the expression level and clinicopathological features (Yasui *et al*, 2004). This study of intrahepatic cholangiocarcinoma indicates that higher expression of *CEACAM6* correlated with lymph node invasion (*P*=0.06), advanced stage of the disease (*P*=0.09), and poorer prognosis (*P*<0.05). Thus, its association between *CEACAM6* expression and poor prognosis in intrahepatic cholangiocarcinoma is similar to that in colorectal cancer or pancreatic cancer.

The reason why *CEACAM6* overexpression is associated with aggressive biological behaviour of cancer cells has not been fully clarified. However, to date several possibilities have been considered. Overexpression of *CEACAM6* is associated with greater resistance to anoikis (Ordonez *et al*, 2000; Ilantzis *et al*, 2002), a subset of apoptosis induced by inadequate or inappropriate cell substrate adhesion, and increased Akt and c-Src kinase activities (Duxbury *et al*, 2004a). Resistance to anoikis is a property of transformed cells that is associated with greater cellular invasive ability and *in vivo* metastatic potential (Yawata *et al*, 1998; Shanmugathasan and Jothy, 2000). Wandering *CEACAM6* overexpressed cells that are resistant to anoikis can reside in the liver and contribute to recurrence. Increased invasiveness following *CEACAM6* overexpression is associated with upregulation of IGF-IR expression and MMP2 expression, which depends upon Akt activation induced by *CEACAM6* overexpression (Duxbury *et al*, 2004c). c-Src-dependent modulation of MMP9 activity contributes significantly to the increased cellular invasiveness induced by *CEACAM6* overexpression (Duxbury *et al*, 2004d). This study demonstrates that the transfectants of the *CEACAM6* gene were more proliferative in MTT assay (Figure 4B and C), had greater invasive abilities in matrigel assay (Figure 5A) and were more resistant to anoikis in anoikis assay (Figure 5B) compared to mock-transfected cells, concurring with the reports described above. Therefore, in intrahepatic cholangiocarcinoma clinical data, these results would be associated with lymphatic invasion and poor prognosis. Furthermore, *CEACAM6* gene silencing reversed the acquired anoikis resistance and inhibited metastatic ability (Duxbury *et al*, 2004a). Suppression of *CEACAM6* expression by siRNA impairs pancreatic adenocarcinoma xenograft growth *in vivo* and improves the survival of tumour-bearing nude mice (Duxbury *et al*, 2004b). Blocking the N and A1B1 domains of *CEACAM5*/*CEACAM6* can impede the metastatic process (Blumenthal *et al*, 2005). Interestingly, another study showed that the *CEACAM6* gene was one of the most overexpressed genes in a side population of cells of hepatocellular carcinoma (Haraguchi *et al*, 2006). Most of the side population of cells are considered to be equal to stem cells that show strong chemoresistance to anticancer drugs. Targeting *CEACAM6* may conceivably be a therapeutic approach for patients with cholangiocarcinoma.

It is difficult to treat cholangiocarcinoma. One reason is that most patients have advanced disease far beyond surgical treatment. The other is that the effects of chemotherapy for cholangiocarcinoma are largely disappointing (Kelley *et al*, 2004; Olnes and Erlich, 2004; Khan *et al*, 2005). 5-Fluorouracil (5-FU) as a single therapy or in combination with other drugs has been studied extensively for cholangiocarcinoma. Although many of these trials were small and uncontrolled, there was an overall response rate of 0–40% and median survival of 2–12 months. With respect to gemcitabine, response rates of 8–60% and median overall survival of 6.3–16 months have been reported. These rates were not changed even when gemcitabine was used in combination with other agents (Thongprasert, 2005). Our study within cholangiocarcinoma cell lines demonstrated that *CEACAM6* overexpressed cells were more chemoresistant to gemcitabine than mock-transfected cells and suppression of *CEACAM6* expression by *CEACAM6* siRNA increased chemosensitivity to gemcitabine. One possible explanation of the relationship between overexpression of *CEACAM6* and gemcitabine resistance is that *CEACAM6* overexpression may protect cells from cytochrome c-induced caspase 3 activation and apoptosis via Akt or c-Src activation. This cytoprotective pathway may contribute to gemcitabine chemoresistance (Duxbury *et al*, 2004e).

In conclusion, this study provided important information that overexpression of *CEACAM6* expression may become a prognostic marker and a chemoresistant indicator for gemcitabine in patients with cholangiocarcinoma. *CEACAM6* could play an important role in cholangiocarcinoma. Further studies in greater numbers of patients should lead to a final conclusion.

## Figures and Tables

**Table 1:** CEACAM6 gene expression and the clinicopathological features of twenty-three patients with intrahepatic cholangiocarcinoma

	**CEACAM6/GAPDH**		
	**High expression group**	**Low expression group**	
	**⩾2 (*n*=10)**	**<2 (*n*=13)**	***P*-value**
Age	64.0±3.9	61.3±3.5	0.61
*Gender*			
Male	7	10	0.54
Female	3	3	
Tumour size	6.3±0.7	5.1±0.6	0.21
			
*Lymph node metastasis*			
Absent	5	10	0.18
Present	5	3	
			
*Lymphatic invasion*			
Absent	3	9	0.06
Present	7	4	
			
*Vascular invasion*			
Absent	2	4	0.46
Present	8	9	
			
*Perineural invasion*			
Absent	4	9	0.16
Present	6	4	
			
*Histology*			
Moderate	8	6	0.11
Poor	2	7	
			
*Stage*			
1, 2, 3	4	10	0.09
4	6	3	
